# Gantenerumab for Early Alzheimer's Disease: An Updated Systematic Review and Meta‐Analysis

**DOI:** 10.1002/brb3.71643

**Published:** 2026-08-16

**Authors:** Muhammad Nouman Javed, Saim Mahmood Khan, Smaher Mustafa, Amna Mujtaba, Hassaan Amin, Naveen Azhar, Abdullah Hameed, Alaa Zayed, Maliha Khalid, Jawairya Muhammad Hussain

**Affiliations:** ^1^ Punjab Medical College Faisalabad Medical University Faisalabad Pakistan; ^2^ Karachi Medical and Dental College Karachi Pakistan; ^3^ Allama Iqbal Medical College Lahore Pakistan; ^4^ Dow Medical College Dow University of Health Sciences Karachi Pakistan; ^5^ Sahiwal Medical College Sahiwal Pakistan; ^6^ Department of Medicine, Faculty of Medicine and Health Sciences An‐Najah National University Nablus Palestine; ^7^ Jinnah Sindh Medical University Karachi Pakistan

**Keywords:** early Alzheimer's disease, gantenerumab, monoclonal antibody, amyloid

## Abstract

**Introduction:**

Alzheimer's disease (AD) is a progressive neurodegenerative disorder with limited treatment options. Gantenerumab, a β‐amyloid–targeting monoclonal antibody, has shown mixed clinical results. With new trial data available, this updated systematic review and meta‐analysis re‐evaluates its efficacy and safety in early AD.

**Methods:**

Registered on PROSPERO (CRD420251082463) under PRISMA guidelines, we searched PubMed, Cochrane Library, and Google Scholar from inception to October 2025 for randomized trials in early AD comparing gantenerumab with placebo. A random‐effects model assessed pooled outcomes with heterogeneity (*I* ^2^) and sensitivity analyses. Additionally, meta‐regression and certainty of evidence using Gradepro were performed. Risk of bias was evaluated using the Cochrane tool. An Institutional Review Committee (IRC) and its Ethical Review Board (ERB) approval was not required.

**Results:**

Six trials (3103 participants: 1718 gantenerumab; 1385 placebo) were included. Gantenerumab showed no significant effect on CDR‐SB (MD = −0.07; 95% CI: −0.34–0.20; *p* = 0.62; *I* ^2^ = 16%) but produced small improvements in FAQ (MD = −0.73; 95% CI: −1.30 to −0.17; *p* *=* 0.01; *I* ^2^ = 0%) and Amyloid‐PET score (MD = −45.67; 95% CI: −88.09 to −3.24; *p* *=* 0.03; *I* ^2^ = 99%). While significant statistical improvement was seen in the ADAS‐Cog13 score (MD = −0.95; 95% CI: −1.76 to −0.13; *p* *=* 0.02; *I* ^2^ = 0%), the effect sizes for both FAQ and ADAS‐Cog13 fell below established MCID thresholds. MMSE and ADCS‐ADL showed no significant differences. Safety analysis revealed higher risks of ARIA‐E (RR = 5.51), ARIA‐H (RR = 1.73), and injection site reactions (RR = 2.15), with no differences in other adverse events.

**Conclusion:**

Gantenerumab significantly reduces amyloid and statistically improves select functional (FAQ) and cognitive (ADAS‐Cog13) measures in early AD; however, these changes remain below accepted MCID thresholds, indicating a lack of true clinical meaningfulness. Combined with its failure to enhance global cognition and an increased ARIA risk, routine clinical use is not supported.

## Graphical abstract

This updated systematic review and meta‐analysis shows that gantenerumab significantly reduces cerebral amyloid burden and modestly improves functional and cognitive outcomes in early Alzheimer's disease, but these benefits are accompanied by an increased risk of amyloid‐related imaging abnormalities, highlighting a cautious benefit–risk balance.

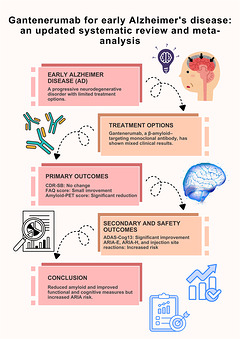

## 1 Introduction

Alzheimer's disease (AD), a chronic, progressive neurodegenerative disorder, is one of the major healthcare challenges of this century (Zheng and Wang 2025). It is a leading cause of dementia globally. According to estimates, by the Year 2050 the number of patients will increase to about 152 million, the greatest rise being projected to occur in developing nations (Janoutová et al. 2021). Individuals with AD experience gradual changes like social withdrawal, anxiety, depression, and disturbed sleep patterns. As the condition progresses, it leads to significant loss of independence and quality of life, thereby posing an important public health concern (Knopman et al. 2021). Despite extensive research, there remains no cure for AD (Office of the Commissioner 2024).

The pathophysiology of AD is driven by a complex cascade of cellular and molecular events that starts several years before the onset of clinical symptoms. Fundamental to this process is the accumulation of β‐amyloid (Aβ) in the extracellular space, forming plaques, and the intracellular aggregation of hyper‐phosphorylated tau into neurofibrillary tangles (NFTs). These changes are coupled with synaptic dysfunction, neuronal loss, and reactive gliosis, leading to progressive neurodegeneration (Jucker and Walker 2023; Yarns et al. 2022). Early changes, such as elevated tau protein in cerebrospinal fluid (CSF) and reduced glucose metabolism in the posterior cingulate cortex, can be detected during the preclinical phase (Sperling et al. 2011). It is generally acknowledged that once an individual enters the earliest stage of this cascade, they are prone to develop symptomatic AD if they survive long enough. Therefore, delaying or halting disease progression in its initial phase remains a primary goal of therapy (Jack et al. 2012; Gauthier 2005). However, treating individuals with no apparent symptoms also raises ethical concerns, particularly regarding the long‐term safety of investigational therapies (Sperling et al. 2012).

Over the years, several drugs have been investigated to prevent or slow the progression of Alzheimer's by targeting Aβ. Early trials of monoclonal antibodies such as solanezumab and bapineuzumab in patients with mild‐to‐moderate AD failed to demonstrate meaningful clinical benefits (Salloway et al. 2014; Doody et al. 2014; Vandenberghe et al. 2016). More recently, interest has shifted to newer anti‐Aβ monoclonal antibodies, and phase III trials are being conducted for donanemab, lecanemab, and gantenerumab, aiming to modify disease progression by targeting various forms of Aβ aggregates (Boxer and Sperling 2023; van Dyck et al. 2023). Gantenerumab, a fully human IgG1 monoclonal antibody administered subcutaneously, has shown promise in targeting aggregated Aβ forms—oligomers, fibrils, and plaques—through microglia‐mediated phagocytosis (Bohrmann et al. 2012). It also facilitates Aβ disaggregation and enhances immune recognition, presenting a comprehensive approach to Aβ clearance. While gantenerumab has demonstrated consistent effects on amyloid biomarkers and neurodegeneration, its impact on clinically meaningful cognitive and functional outcomes remains uncertain (Bateman et al. 2022).

A prior systematic review and meta‐analysis published in 2024 by De Almeida et al. (2024) synthesized evidence from four randomized trials and concluded that gantenerumab did not demonstrate meaningful clinical efficacy and was associated with safety concerns in early AD. However, since then, a number of significant phase III randomized controlled trials (RCTs), including the GRADUATE I and II trials, have reported additional safety and efficacy data, thereby increasing the body of available evidence. An updated review is therefore required to determine whether the expanded evidence influences earlier conclusions regarding the therapeutic value of gantenerumab as the newer trials included larger populations and updated dosing regimens, focusing on clinically relevant endpoints.

Importantly, interpretation of treatment effects in AD requires consideration of minimal clinically important differences (MCIDs), as statistically significant changes may not translate into clinically meaningful benefit. Prior studies suggest that changes on the order of approximately 0.5–1.0 points for the Clinical Dementia Rating–Sum of Boxes (CDR‐SB) and 3–4 points for the Alzheimer's Disease Assessment Scale–Cognitive Subscale (ADAS‐Cog13) are generally considered clinically meaningful, with similar considerations applying to functional measures such as the Functional Activities Questionnaire (FAQ) and Alzheimer's Disease Cooperative Study–Activities of Daily Living (ADCS‐ADL) (Andrews et al. 2019). Without contextualizing effect sizes against these benchmarks, the clinical relevance of observed differences remains difficult to assess.

Accordingly, the objective of the present systematic review and meta‐analysis is to re‐evaluate the efficacy and safety of gantenerumab in early AD using the most up‐to‐date randomized evidence. The primary outcomes are changes in global cognition and function, assessed by the CDR‐SB and FAQ, as well as amyloid burden measured by amyloid positron emission tomography (Amyloid‐PET). Secondary outcomes include additional cognitive and functional measures such as ADAS‐Cog13, Mini‐Mental State Examination (MMSE), and ADCS‐ADL. Safety outcomes, with particular emphasis on amyloid‐related imaging abnormalities: Vasogenic edema (ARIA‐E) and hemosiderin (ARIA‐H), are evaluated to characterize the risk profile of gantenerumab. By integrating newly available data and interpreting results in relation to established MCID thresholds, this study aims to clarify the clinical significance of gantenerumab's effects in early AD.

## 2 Methods

### 2.1 Study Design

A systematic review and meta‐analysis was conducted in accordance with the Preferred Reporting Items for Systematic Reviews and Meta‐Analysis (PRISMA) guidelines (Liberati et al. 2009). The prescribed procedures for this study have been formally documented and registered in the PROSPERO (International Prospective Register of Systematic Reviews) database, with the protocol number designated as CRD420251082463.

### 2.2 Data Sources and Search Strategy

We extensively searched core databases, including PubMed and the Cochrane Library, for relevant literature published from inception up till October 2025. Additionally, Google Scholar was searched as a supplementary source to capture gray literature and ensure comprehensive coverage. To ensure search reproducibility despite Google Scholar's dynamic ranking algorithms, this search was executed on a single day (October 24, 2025), sorted by relevance, and restricted to screening the first 200 hits, which yielded 189 unique records for initial evaluation. Additional trials were searched through ClinicalTrials.gov and Medrxiv.org. Target search terms included keywords and medical subject headings (MeSH) where applicable, such as “Alzheimer's Disease,” “Alzheimer's Dementia,” “Early Onset Alzheimer's Disease,” “asymptomatic,” and “gantenerumab.”

Where accessible, each database's restricted vocabulary was used in searches along with free‐text words, which were tailored to each data library individually. Boolean operators “AND” were used to combine different concept groups, while “OR” was used to include synonyms within the same concept group. The articles published in English were considered. Furthermore, articles from the reference citations were further evaluated to ensure more qualifying results.

### 2.3 Study Selection and Inclusion Criteria

**FIGURE 1:**
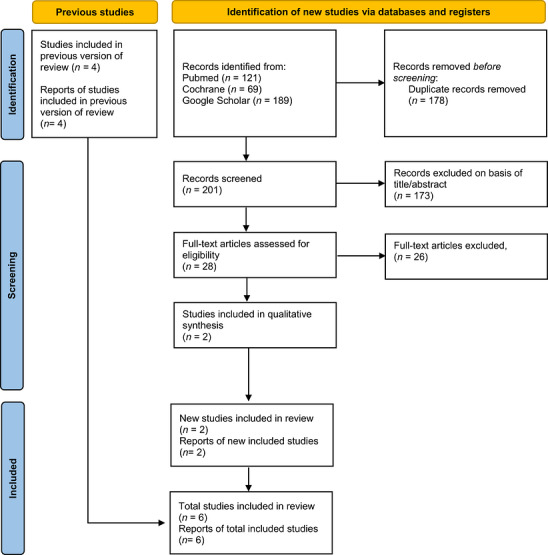
Preferred reporting items for systematic review and meta‐analysis. PRISMA flow diagram of study screening and selection.

The articles were selected based on PICOS (participants, intervention, comparison, outcomes, and study design) concept. The duplicates were identified and removed using Endnote X7 (Clarivate Analytics, Thomson Reuters Corporation). Studies were selected according to the following inclusion criteria: (1) Patients diagnosed with early‐stage AD. This includes patients with Mild Cognitive Impairment (MCI) or mild dementia due to AD, defined by the National Institute on Aging‐Alzheimer's Association (NIA‐AA) core clinical criteria for probable AD dementia or prodromal AD, and confirmed by evidence of cerebral beta‐amyloid pathology via CSF or Amyloid‐PET scan (Sperling et al. 2011). (2) The studies evaluating the efficacy of gantenerumab, (3) studies reporting at least one outcome of interest, and (4) studies using placebo as control group. Studies using multidrug therapy or with no outcome of interest, letters, conference papers, and case reports were excluded. The details of the research and selection procedure are depicted in the PRISMA flowchart (Figure 1).

### 2.4 Data Extraction and Outcomes

Two authors independently extracted data from the included articles, capturing author names, publication years, participant numbers, mean ages, and sex distribution. For missing or unclear data, corresponding authors were contacted through email. The primary outcomes were CDR‐SB, FAQ, and Amyloid‐PET score. The secondary outcomes include MMSE score, ADCS‐ADL score, and ADAS‐Cog13 score. Safety outcomes—including ARIA‐E, ARIA‐H, headache, falls, injection site events, back pain, diarrhea, dizziness, urinary tract infections (UTIs), and nasopharyngitis—were also analyzed.

### 2.5 Statistical Analysis and Quality Assessment

All statistical analysis was conducted using RevMan (version 5.4.1). OpenMeta [Analyst] (version 5.26.14) was used for meta‐regression analyses. Dichotomous outcomes were reported as Mantel–Haenszel risk ratios (RRs), while continuous data were presented as mean differences (MDs) with 95% confidence intervals (CIs), using the inverse variance method. A random effects model was applied throughout due to expected variability among studies. Statistical significance was set at a *p* value of < 0.05, and findings are illustrated using forest plots. Heterogeneity was evaluated using Higgins’ *I* ^2^ statistic, with values of 25%–50% considered mild, 50%–75% moderate, and over 75% as substantial. The risk of bias was assessed independently by two authors using the Revised Cochrane risk‐of‐bias tool for randomized trials (ROB 2) (Sterne et al. 2019). Additionally, the quality of evidence of each outcome was assessed using GRADEpro (McMaster University, Evidence Prime Inc. 2024). Disagreements were resolved through discussion among the research team to ensure consensus.

Potential publication bias was visually evaluated by constructing funnel plots for the outcomes containing the maximum number of trials, specifically the primary endpoint (CDR‐SB score; *n* = 5 trials) and the key secondary cognitive endpoint (ADAS‐Cog13 score; *n* = 5 trials). Due to the limited number of component trials (*n* < 10), these plots were primarily interpreted qualitatively alongside a rigorous evaluation of database and trial registry search outputs, accounting for the low statistical power inherent to asymmetry assessments in small datasets. Further, to assess the robustness of the findings and assess heterogeneity, a leave‐one‐out sensitivity analysis was conducted (Higgins and Thompson 2002). This involved sequentially excluding each study from the meta‐analysis to observe its impact on heterogeneity (*I* ^2^) and the pooled effect estimate.

## 3 Results

### 3.1 Search Results and Baseline Characteristics

As outlined in the PRISMA flow diagram (Figure 1), the initial database search identified 379 records. Following the removal of duplicates and screening by title and abstract, 28 articles were selected for full‐text review. Following detailed evaluation, 26 studies were excluded for reasons such as inappropriate study design (e.g., reviews and case reports), lack of placebo control, non‐AD populations, or insufficient data for extraction. Ultimately, six studies met the inclusion criteria, encompassing a total of 3103 participants. Among them, 1718 individuals received subcutaneous gantenerumab, while 1385 were assigned to placebo groups. All included participants were diagnosed with either early‐stage or prodromal AD.

Of the six included studies, one (DIAN‐TU) enrolled participants with either prodromal or mild AD, while all the remaining trials included only patients with mild disease. For the purpose of this meta‐analysis, only the data corresponding to participants with mild AD from the DIAN‐TU study were extracted and analyzed, ensuring homogeneity across the included studies. This approach minimized clinical heterogeneity and allowed for a more accurate comparison of gantenerumab efficacy and safety within a uniform disease stage. Key baseline characteristics from the included studies are summarized in Table 1.

**TABLE 1:** Baseline characteristics of included studies.

	No. patients (*N*)		Mean age, years (mean ± SD)		Female sex (*N*) (%)		Mean CDR‐SB (mean ± SD)		Mean MMSE (mean ± SD)	
Study, year	Gantenerumab	Placebo	Gantenerumab	Placebo	Gantenerumab	Placebo	Gantenerumab	Placebo	Gantenerumab	Placebo
DIAN‐TU et al. 2021	52	40	46.0 ± 10.8	44.2 ± 9.6	21 (40)	22 (55)	1.33 ± 2.08	1.43 ± 1.87	27.10 ± 3.45	26.68 ± 3.97
GRADUATE I et al. 2023	499	485	71.1 ± 7.9	72.1 ± 7.8	290 (58.1)	255 (52.6)	3.71 ± 1.67	3.71 ± 1.57	23.5 ± 3.3	23.6 ± 3.0
GRADUATE II et al. 2023	498	477	71.6 ± 7.8	71.8 ± 7.4	288 (57.8)	285 (59.7)	3.67 ± 1.61	3.52 ± 1.54	23.6 ± 3.1	23.8 ± 3.2
SCarlet RoAD et al. 2017	Low Dose: 271 High Dose: 260	266	Low Dose: 70.3 ± 7.0 High Dose: 71.3 ± 7.1	69.5 ± 7.5	NA	NA	Low Dose: 2.2 ± 1.0 High Dose: 2.0 ± 0.9	2.1 ± 1.0	Low Dose: 25.7 ± 2.3 High Dose: 25.7 ± 2.2	25.7 ± 2.1
Asada et al. 2025	Low dose: 17 High dose: 34	16	Low dose: 74.1 ± 5.3 High dose: 71.3 ± 7.7	72.3 ± 8.0	Low dose: 12 (70.6) High dose: 19 (55.9)	12 (75.0)	Low dose: 3.47 ± 1.66 High dose: 3.43 ± 1.05	2.78 ± 1.09	Low dose: 23.4 ± 3.5 High dose: 23.2 ± 3.0	23.8 ± 2.7
Neve et al. 2024	108	117	71.0 ± 9.3	71.8 ± 8.1	61 (56.5)	69 (59.0)	5.66 ± 2.68	5.86 ± 2.87	19.56 ± 4.79	19.78 ± 5.21

Abbreviations: CDR‐SB, Clinical Dementia Rating‐Sum Of Boxes; MMSE, Mini‐Mental State Examination; NA, not available; SD, standard deviation.

### 3.2 Quality Assessment

The risk of bias analysis of all included studies is demonstrated in Figure S1. Four of the six included RCTs (DIAN‐TU 2021, GRADUATE I 2023, GRADUATE II 2023, SCarlet RoAD 2017) were rated low risk across all RoB 2 domains. Asada et al. (2025) showed high risk in randomization and low risk for other domains. Neve et al. (2024) showed high risk for missing outcome data along with some concerns in the randomization process. Thus, 67% of trials were low risk overall, while 33% carried significant bias in key domains, warranting caution in interpreting pooled results.

### 3.3 Primary Outcomes (Clinical Scores)

For all clinical outcomes reported below, the forest plot legends display an inverted treatment direction. In these measures, negative MDs indicate less clinical deterioration, meaning values shifted to the left (negative) favor gantenerumab, not placebo.

#### 3.3.1 CDR‐SB Score

**FIGURE 2:**
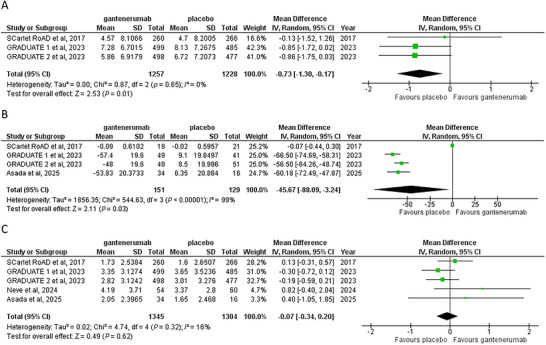
Primary outcomes. FAQ score changes from baseline (A), Amyloid‐PET score changes from baseline (B), and CDR‐SB score changes from baseline (C).

Five RCTs, including a total of 2649 participants (1345 gantenerumab; 1304 placebo), reported cognitive function using the CDR‐SB. The pooled analysis revealed no significant difference between groups (MD = −0.07; 95% CI: −0.34–0.20; *p* = 0.62; *I* ^2^ = 16%; Figure 2C; CDR‐SB score). Although the combined effect was slightly negative, indicating a trend toward less clinical decline with gantenerumab, this did not reach statistical significance.

#### 3.3.2 FAQ Score

Functional ability was evaluated using the FAQ score in three studies comprising 2485 patients (1257 gantenerumab; 1228 placebo). A small but statistically significant improvement was observed in the gantenerumab group compared to placebo (MD = −0.73; 95% CI: −1.30 to −0.17; *p* = 0.01; *I* ^2^ = 0%; Figure 2A; FAQ score). As lower FAQ scores reflect better functional performance, the negative MD favors gantenerumab. However, this absolute difference of −0.73 points falls below accepted real‐world MCID benchmarks for functional independence, indicating that this statistical significance may not translate into perceptible day‐to‐day benefits for patients.

#### 3.3.3 Amyloid‐PET Score

Changes in brain amyloid burden were reported in four studies involving 280 participants. The analysis demonstrated a significant reduction in amyloid levels with gantenerumab compared to placebo (MD = −45.67; 95% CI: −88.09 to −3.24; *p* = 0.03; *I* ^2^ = 99%; Figure 2B; Amyloid‐PET score). The strong negative direction of effect indicates a clear amyloid‐lowering trend for gantenerumab; however, the extreme statistical heterogeneity (*I* ^2^ = 99%) demands an exceptionally cautious interpretation, signaling that the absolute magnitude and velocity of plaque clearance vary profoundly across individual trials and cannot be generalized as a uniform effect size.

### 3.4 Secondary Outcomes (Clinical Scores)

As noted, in all secondary outcomes, negative MD values indicate a greater benefit of gantenerumab due to less decline or improvement in the respective domains.

#### 3.4.1 MMSE Score, ADCS‐ADL Score, and ADAS‐Cog13 Score

**FIGURE 3:**
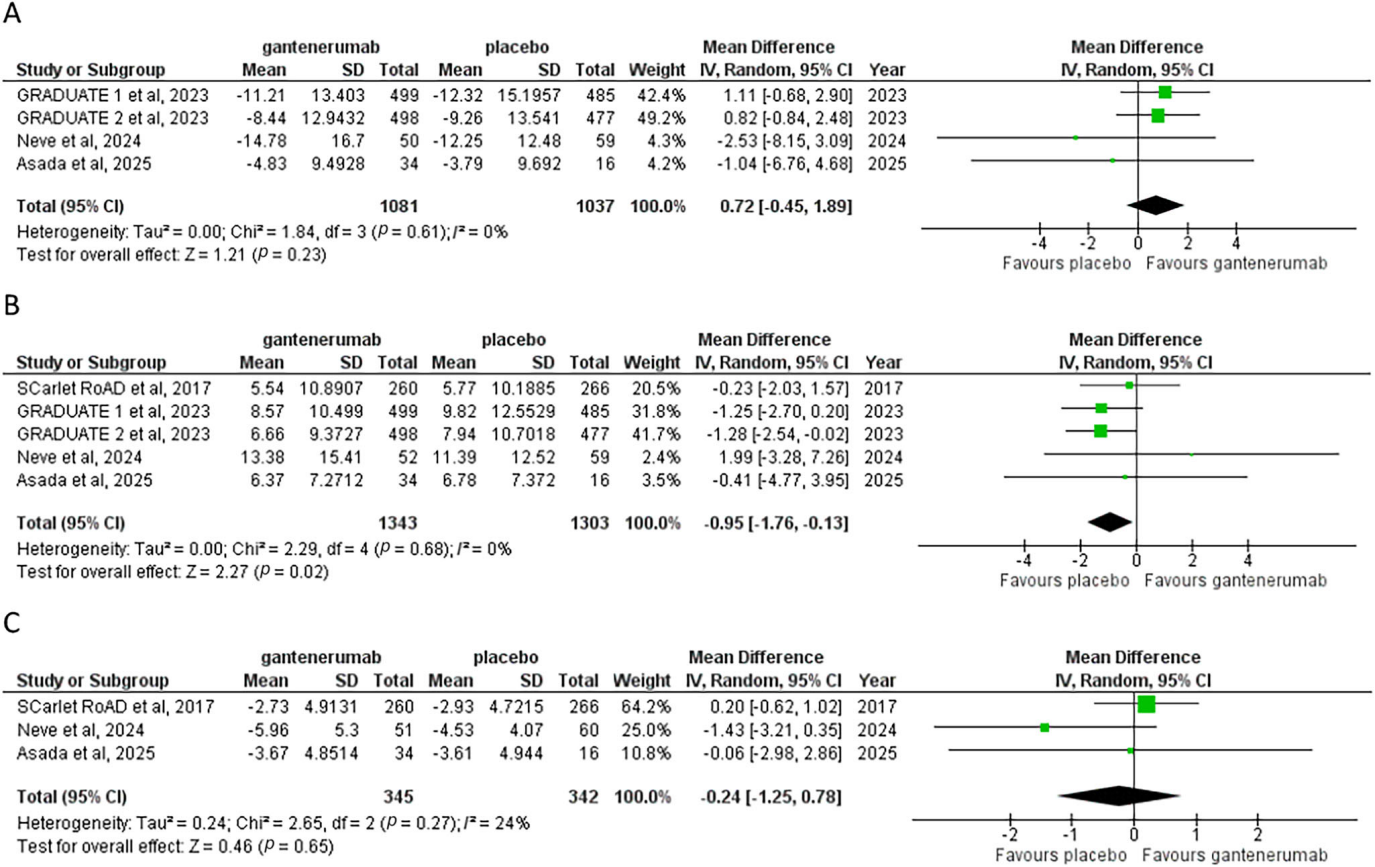
Secondary outcomes. ADCS‐ADL score changes from baseline (A), and ADAS‐Cog13 score changes from baseline (B), MMSE score changes from baseline (C).

Among secondary cognitive measures, the MMSE score, reported in three studies, showed no significant difference between groups (MD = −0.24; 95% CI: −1.25–0.78; *p* = 0.65; *I* ^2^ = 24%; Figure 3C; MMSE score). Similarly, no significant difference was found in functional status using the ADCS‐ADL across four studies (MD = 0.72; 95% CI: −0.45–1.89; *p* = 0.23; *I* ^2^ = 0%; Figure 3A; ADCS‐ADL score). A small but statistically significant cognitive benefit was observed in the ADAS‐Cog13 based on five studies (MD = −0.95; 95% CI: −1.76 to −0.13; *p* *=* 0.02; *I* ^2^ = 0%; Figure 3B; ADAS‐Cog13 score). Crucially, this absolute MD (−0.95 points) is substantially lower than the widely accepted MCID threshold of 3–4 points for the ADAS‐Cog13 subscale, demonstrating that this isolated cognitive statistical advantage lacks actual clinical relevance.

### 3.5 Safety Outcomes

#### 3.5.1 ARIA‐E and ARIA‐H

**FIGURE 4:**
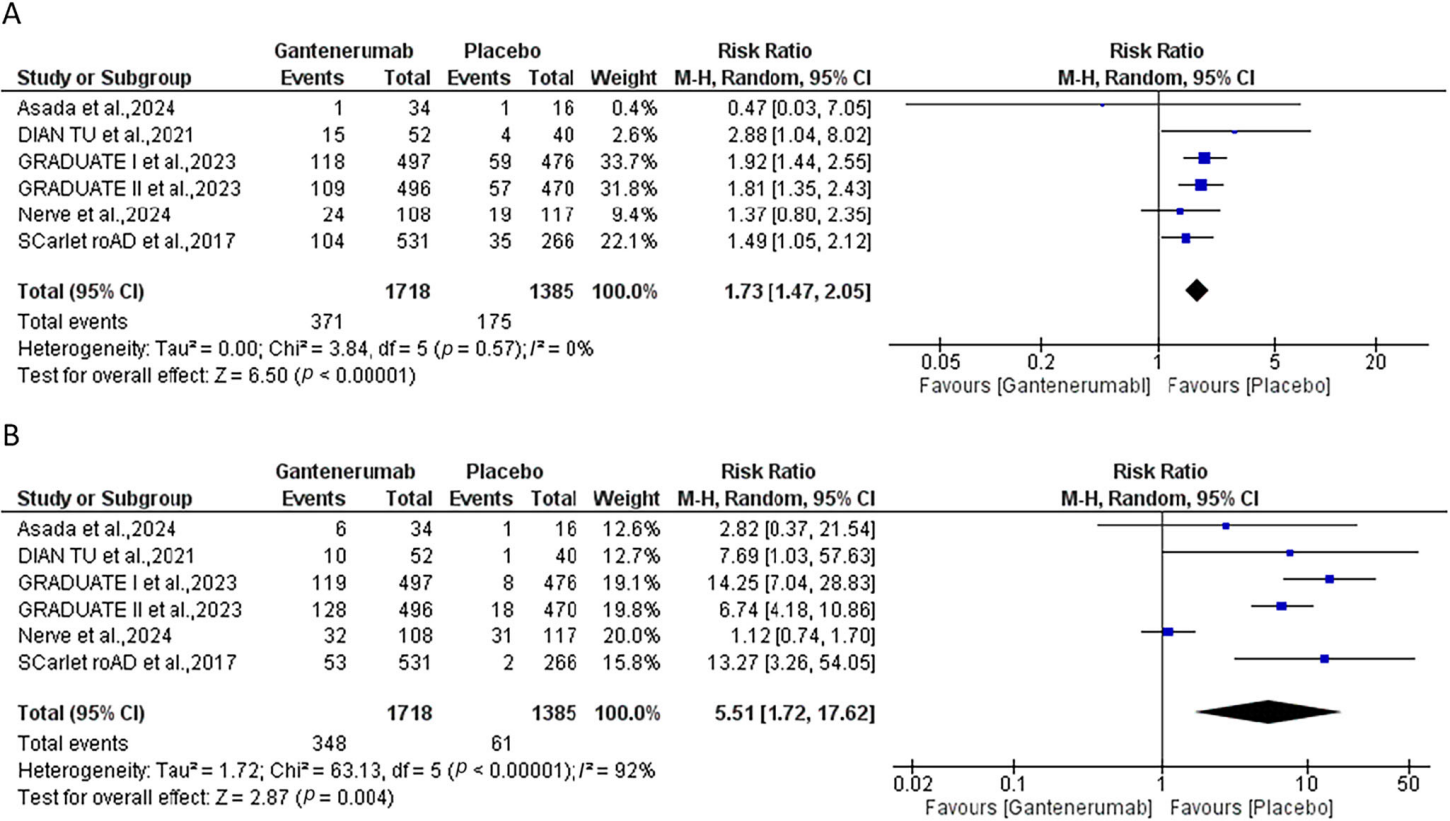
Safety outcomes. Forest plot comparing the occurrence of ARIA‐H (A) and ARIA‐E (B) between groups.

In comparison with placebo, the occurrence of ARIA‐E and ARIA‐H was both significantly higher in the gantenerumab group. For ARIA‐E (RR: 5.51; 95% CI: 1.72–17.62; *p* = 0.004; *I* ^2^ = 92%; Figure 4B; ARIA‐E). For ARIA‐H (RR: 1.73; 95% CI: 1.47–2.05; *p* < 0.00001; *I* ^2^ = 0%; Figure 4A; ARIA‐H). These findings confirm that gantenerumab is associated with a significantly increased risk of both ARIA‐E and ARIA‐H, with a notably higher effect size observed for ARIA‐E. The low heterogeneity for ARIA‐H supports consistency across included studies. In contrast, the substantial heterogeneity for ARIA‐E (*I* ^2^ = 92%) necessitates a cautious interpretation; while the risk is consistently and significantly elevated across the board, the wide variation in RRs indicates that the precise likelihood of developing vasogenic edema is highly volatile and likely dependent on cohort‐specific factors rather than representing a fixed, predictable universal risk.

### 3.6 Gantenerumab Adverse Events

A total of eight specific adverse events were assessed to evaluate the safety of gantenerumab compared to placebo. A statistically significant difference was observed only for injection site reactions, which were more frequent in the gantenerumab group (RR = 2.15; 95% CI: 1.34–3.46; *p* = 0.002; *I* ^2^ = 82%; Figure 5A; injection site reactions). This suggests more than a twofold increased risk, though substantial heterogeneity was present, indicating variability across studies that may reflect differences in dose, duration, or patient populations.

FIGURE 5

**Figure:**
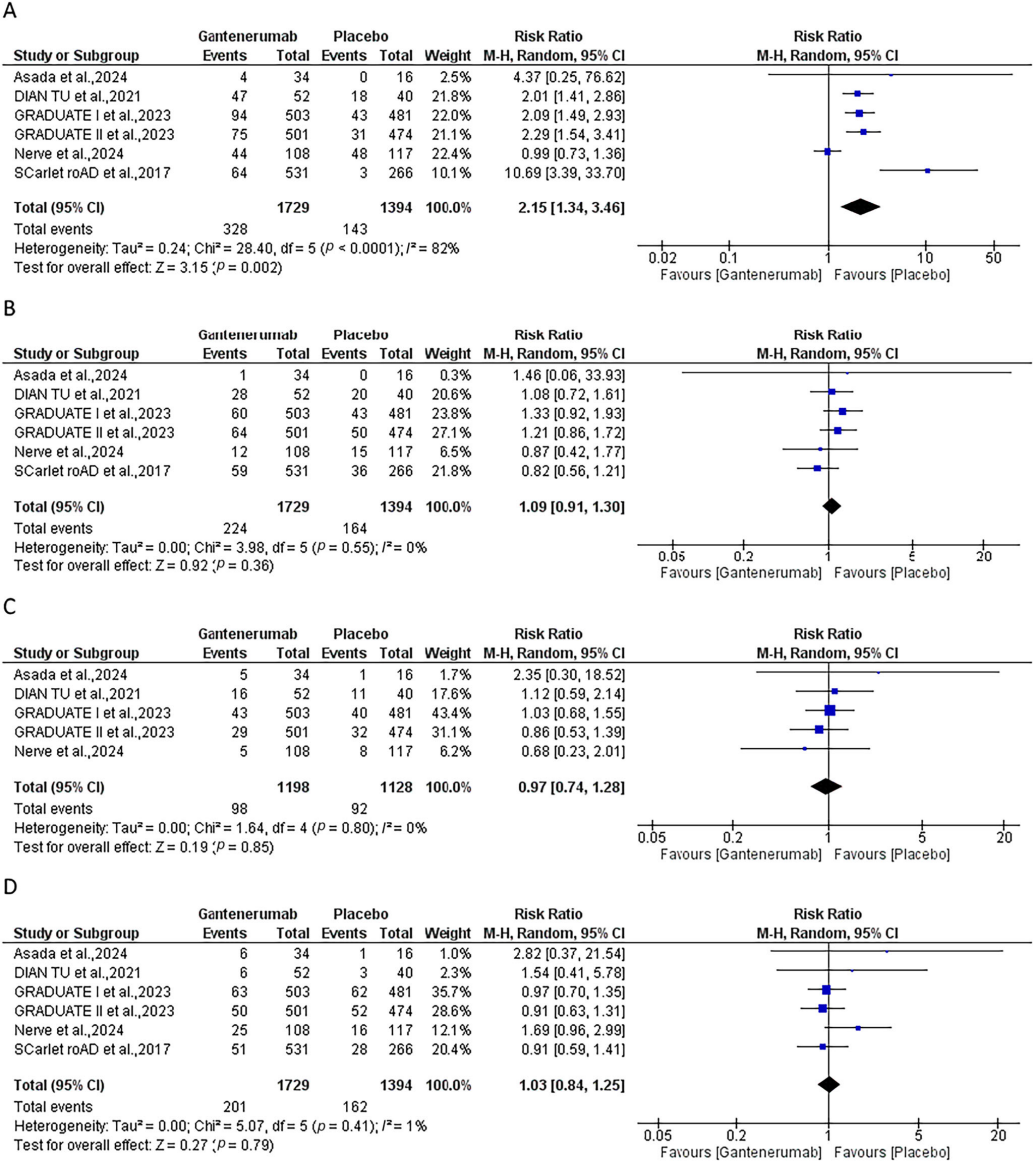
Forest plots comparing the occurrence of AEs between groups: injection site events (A), headache (B), back pain (C), fall (D), dizziness (E), diarrhea (F), urinary tract infections (G), and nasopharyngitis (H).

**Figure:**
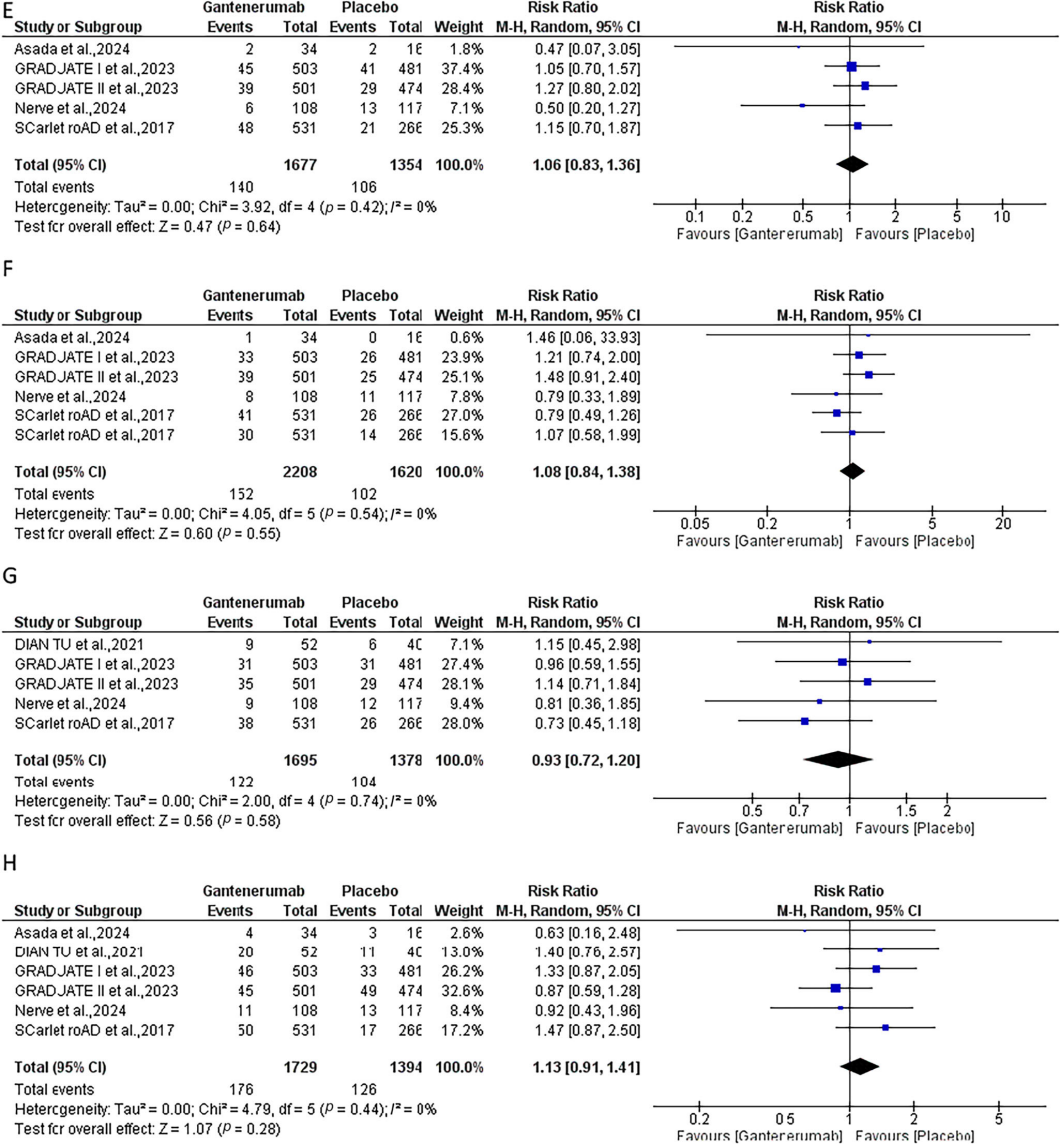

No statistically significant differences were observed for any of the remaining adverse events. Headache occurred at similar rates between groups (RR = 1.09; 95% CI: 0.91–1.30; *p* = 0.36; *I* ^2^ = 0%; Figure 5B; headache), as did back pain (RR = 0.97; 95% CI: 0.74–1.28; *p* = 0.85; *I* ^2^ = 0%; Figure 5C; back pain), fall (RR = 1.03; 95% CI: 0.84–1.25; *p* = 0.79; *I* ^2^ = 1%; Figure 5D; fall), dizziness (RR = 1.06; 95% CI: 0.83–1.36; *p* = 0.64; *I* ^2^ = 0%; Figure 5E; dizziness), diarrhea (RR = 1.08; 95% CI: 0.84–1.38; *p* = 0.55; *I* ^2^ = 0%; Figure 5F; diarrhea), UTIs (RR = 0.93; 95% CI: 0.72–1.20; *p* = 0.58; *I* ^2^ = 0%; Figure 5G; UTIs), and nasopharyngitis (RR = 1.13; 95% CI: 0.91–1.41; *p* = 0.28; *I* ^2^ = 0%; Figure 5H; nasopharyngitis). Although some events, such as nasopharyngitis and dizziness, were numerically higher in the gantenerumab group, none reached statistical significance. Across all non‐significant outcomes, the 95% CIs crossed the line of no effect, and heterogeneity was negligible.

### 3.7 Meta‐Regression of ARIA‐E and ARIA‐H Versus Baseline CDR‐SB

**FIGURE 6:**
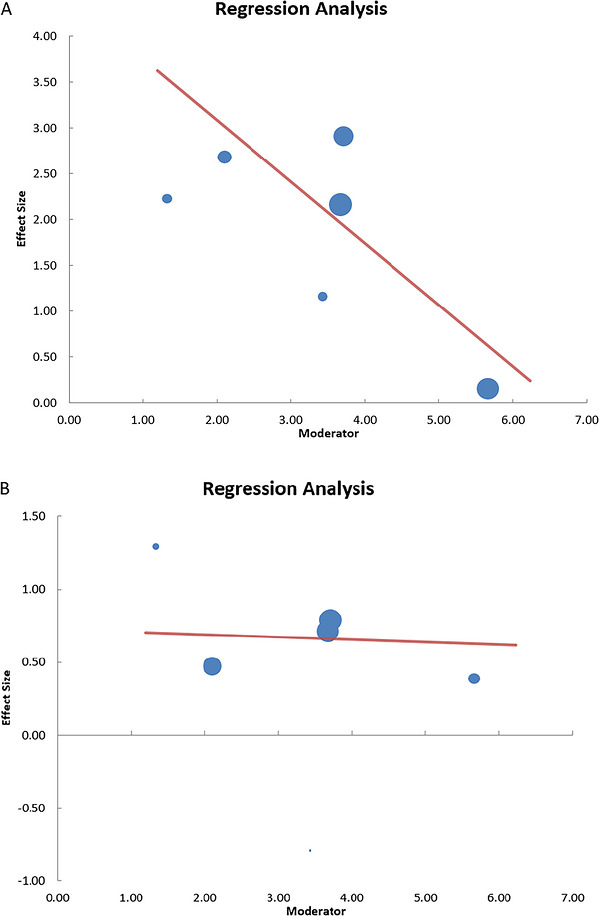
Meta‐regression bubble plot of ARIA‐E versus baseline CDR‐SB score (A) and meta‐regression bubble plot of ARIA‐H versus baseline CDR‐SB score (B).

In a random‐effects meta‐regression using baseline CDR‐SB score as a continuous moderator, higher CDR‐SB was associated with significantly lower odds of ARIA‐E (log‐odds = –0.67, SE = 0.22; 95% CI [–1.24, –0.10]; *Z* = –3.03; *p* = 0.002; Figure 6A). By contrast, baseline CDR‐SB showed a small, non‐significant negative relationship with the odds of ARIA‐H (log‐odds = –0.02, SE = 0.10; 95% CI [–0.28, 0.24]; *Z* = –0.17; *p* = 0.862; Figure 6B), suggesting no evidence that initial disease severity predicts ARIA‐H risk.

### 3.8 Sensitivity Analysis

**FIGURE 7:**
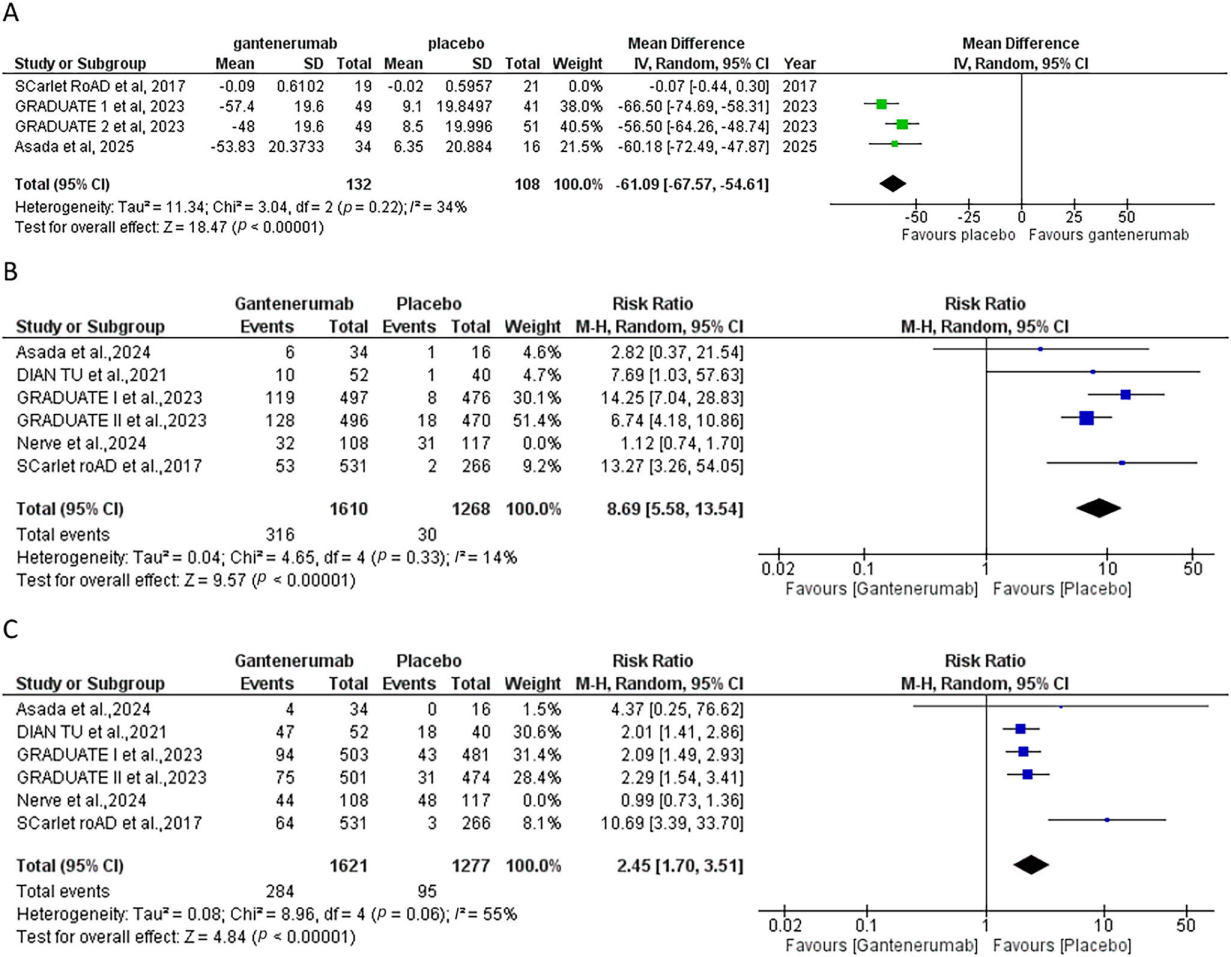
Sensitivity analysis. Amyloid PET Score (A), ARIA‐E (B), and injection site (C).

The original analysis demonstrated substantial heterogeneity in Amyloid‐PET score (*I* ^2^ = 99%; Figure 2B; Amyloid‐PET score). Notably, the exclusion of the study by SCarlet RoAD (2017) reduced the heterogeneity dramatically from 95% to 34%, indicating that this study was the primary contributor to the between‐study variability. In contrast, exclusion of any of the remaining three studies resulted in minimal to no change in heterogeneity, suggesting they had negligible impact on the overall variability of the results. Similarly for the safety outcome ARIA‐E and the adverse event injection site, the Neve et al. (2024) study contributed to significant heterogeneity. By excluding this study, heterogeneity reduced from 92% to 14% in ARIA‐E (Figure 7B; sensitivity analysis) and 82% to 55% in the injection site (Figure 7C; sensitivity analysis).

### 3.9 Certainty of Evidence

The certainty of evidence was predominantly moderate across efficacy outcomes as reported in Figure S3. Specifically, the evidence for changes in CDR‐SB, FAQ, ADCS‐ADL, and ADAS‐Cog13 scores and amyloid‐PET reduction was rated as moderate certainty, while MMSE was rated low certainty. Although the current findings suggest a consistent direction of benefit, the results indicate that further research may have an important impact on the estimate of effect. For safety outcomes, the certainty of evidence varied: ARIA‐H and injection site events were supported by high‐certainty evidence, reflecting robust and consistent estimates across trials. In contrast, the evidence for ARIA‐E, headache, falls, nasopharyngitis, dizziness, UTIs, back pain, and diarrhea was judged to be of moderate certainty, largely due to imprecision or variability in effect estimates. Overall, the clinical efficacy and safety outcomes are supported by moderate‐certainty evidence, warranting cautious interpretation and further confirmatory research.

### 3.10 Publication Bias

**FIGURE 8:**
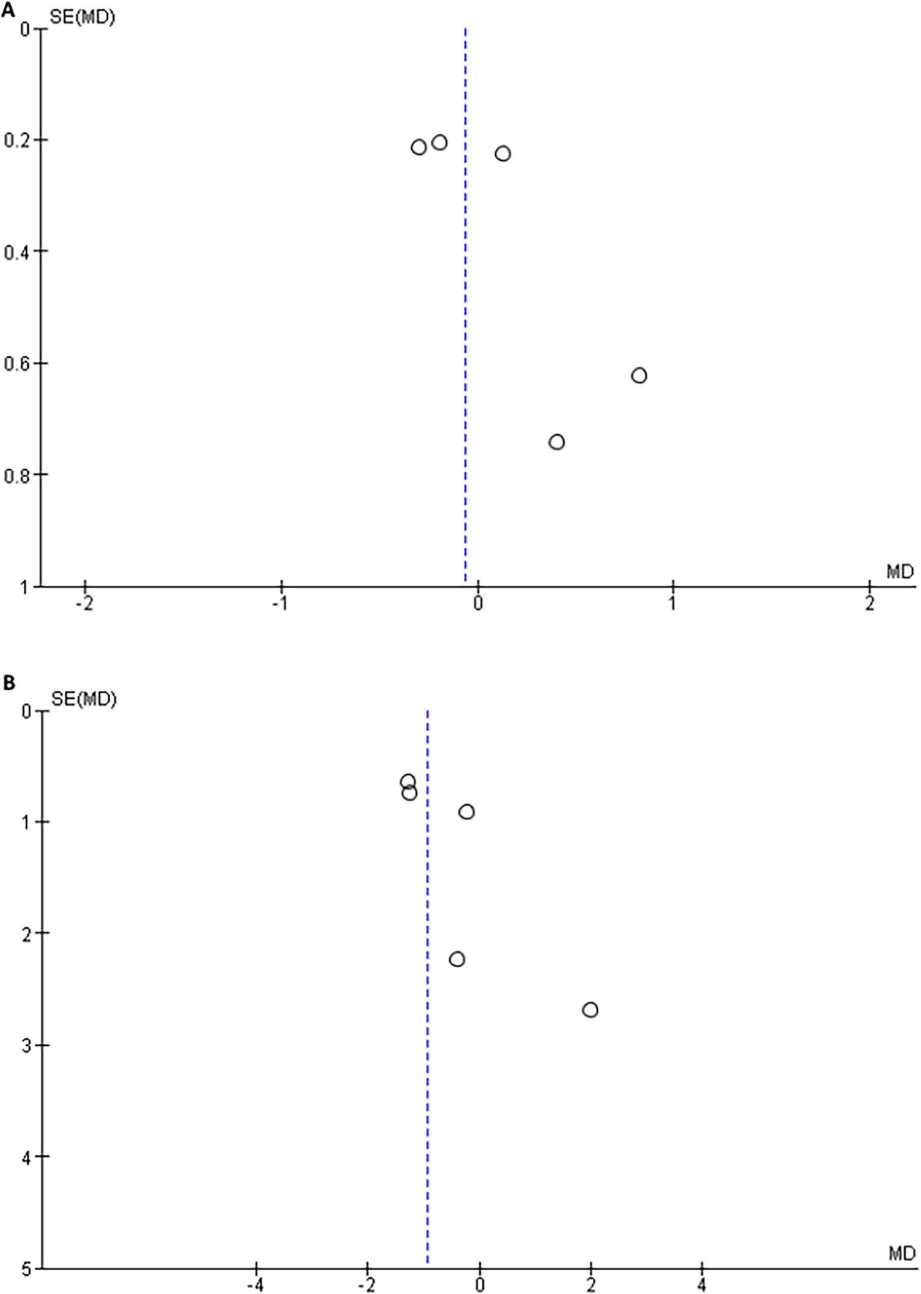
Funnel plot showing publication bias in (A) CDR‐SB score and (B) ADAS‐Cog13 score.

To evaluate the presence of potential publication or selective reporting bias, funnel plots were generated for the primary cognitive endpoint (CDR‐SB) and the secondary cognitive endpoint showing a statistical signal (ADAS‐Cog13), each drawing from five eligible trials (Figure 8). Visual inspection of the funnel plot for the CDR‐SB score (Figure 8A) and the ADAS‐Cog13 score (Figure 8B) revealed a generally symmetric, inverted funnel distribution. In both configurations, small and large studies were dispersed relatively evenly on either side of the pooled global MD line. This symmetric clustering suggests that the overall pooled estimates are unlikely to be heavily distorted by the selective non‐publication of small‐sample negative datasets, though these visual diagnostics remain bounded by the low statistical power typical of small trial pools.

## 4 Discussion

This updated meta‐analysis builds upon previous systematic evidence (De Almeida et al. 2024) by compiling data from multiple RCTs to re‐evaluate the clinical effectiveness and safety profile of gantenerumab in early‐stage AD. Our pooled findings reveal a stark therapeutic paradox: while subcutaneous gantenerumab achieves robust biological target engagement by significantly clearing cerebral amyloid plaques, this biomarker success fails to translate into widespread, clinically meaningful improvements in global cognition or daily functioning (Digma et al. 2024). Moreover, this modest clinical impact is accompanied by a pronounced increase in treatment‐limiting adverse events, particularly ARIA. Consequently, these data underscore the ongoing uncertainty surrounding the clinical utility of isolated amyloid clearance and emphasize the critical need for standardized, long‐term trials to evaluate anti‐amyloid therapies through the lens of established clinical benchmarks.

### 4.1 Efficacy on Primary Cognitive and Functional Endpoints

The pooled analysis of five RCTs involving 3103 participants demonstrated no statistically significant difference in global cognitive decline on the CDR‐SB between the gantenerumab and placebo groups, indicating that the drug does not meaningfully alter progression. This lack of clinical efficacy stands in sharp contrast to the drug's clear biological activity. As a fully human IgG1 antibody engineered to target aggregated Aβ fibrils and plaques, gantenerumab binds with high affinity and specificity to clear pathological deposits from the brain (Bateman et al. 2022). This biological engagement is evidenced by our pooled analysis of 280 participants across four studies showing a marked reduction in brain amyloid burden, including reductions of −66.44 and −56.46 centiloids and up to 26.8% of participants achieving amyloid‐negative status in the GRADUATE trials. Nevertheless, the massive heterogeneity (*I* ^2^ = 99%) observed in our pooled PET data introduces an element of uncertainty that requires cautious clinical interpretation, as differences in baseline plaque burden, treatment durations, and tracking methods across the literature complicate the projection of a standardized biological response.

This profound disconnect highlights the ongoing debate regarding the amyloid cascade hypothesis (Tran and Tran 2025), suggesting that clearing established plaques in isolation may be insufficient to yield robust clinical improvements once clinical symptoms manifest, potentially pointing to downstream tau pathology as the more direct driver of localized neurodegeneration and visible clinical symptoms (Bilgel et al. 2022; Villegas et al. 2021). Furthermore, while cumulative anti‐amyloid trial data showed a MD of only −0.08 for the CDR‐SB, studies examining MCIDs establish that a change of 0.5 points or greater is the minimum threshold for clinical relevance. Because the 95% CIs cross zero, the observed effects are too small to be clinically meaningful, confirming that biological target engagement did not translate into definitive global cognitive improvements.

In terms of functional outcomes, three studies assessing 2485 participants reported a small but statistically significant improvement in functional ability via the FAQ score. However, this functional signal must be interpreted cautiously. Functional decline typically progresses more slowly and variably than cognitive decline during the MCI or early dementia stages targeted by these trials, where daily living activities remain relatively preserved. Because functional decline tends to accelerate later in the disease course, the limited duration of these phase III trials may fail to fully capture meaningful longitudinal trajectories (De Almeida et al. 2024; Smith et al. 2023). This indicates that while early intervention may offer a subtle stabilizing effect, longer follow‐up is required to confirm definitive functional utility.

### 4.2 Efficacy on Secondary Biomarker and Exploratory Cognitive Outcomes

The secondary outcomes further reinforce this trend of constrained clinical utility. Both global cognitive screening (MMSE) and broad functional status (ADCS‐ADL) demonstrated no significant differences between the treatment and control groups, with low heterogeneity indicating high consistency across the underlying trial data. Interestingly, a small but statistically significant benefit was isolated on the ADAS‐Cog13 subscale. Rather than signaling a broader clinical breakthrough, this slight cognitive advantage likely reflects the unique sensitivity of the ADAS‐Cog13 to subtle cognitive changes that global metrics miss. Given the completely flat outcomes observed across the concurrent MMSE, CDR‐SB, and ADCS‐ADL scales, the real‐world impact of this minor shift on a patient's daily quality of life and independent daily functioning remains highly limited.

### 4.3 Safety Profile and Clinical Significance of ARIA

ARIA‐E and ARIA‐H represent major safety concerns consistently identified in the MRI‐monitored cohorts. The meta‐analysis revealed a more than fivefold increase in the risk of ARIA‐E compared to placebo. Mechanistically, this vasogenic edema is driven by temporarily increased vascular permeability occurring when gantenerumab interacts with pre‐existing Aβ deposits within cerebral blood vessel walls (Salloway et al. 2024; Agarwal et al. 2023). This disruption occurs because AD patients have baseline amyloid deposition in vessel walls, which disrupts smooth muscle cell integrity and compromises vascular structure (Agarwal et al. 2023; Prior et al. 1996). This interaction triggers an enhanced mobilization of Aβ that causes transient increases in vascular permeability (Salloway et al. 2024; Agarwal et al. 2023) and overwhelms the perivascular drainage system—the brain's primary route for clearing interstitial fluid and soluble proteins like Aβ (Hawkes et al. 2014; Weller et al. 2008). Consequently, proteinaceous fluid extravasates into the brain parenchyma and sulci, manifesting as vasogenic edema (Hawkes et al. 2014; Ullah and Lee 2023). This process is further exacerbated because gantenerumab activates microglia through Fc gamma receptor‐mediated phagocytosis of Aβ (Bateman et al. 2022; Piazza et al. 2022), releasing pro‐inflammatory mediators that compromise blood‐brain barrier (BBB) integrity in regions showing spatial co‐localization with microglial activation (Piazza et al. 2022).

Similarly, gantenerumab was associated with a highly consistent, significant increase in ARIA‐H events across trials. This risk stems from the physical disruption of structurally compromised vessel walls affected by pre‐existing cerebral amyloid angiopathy (CAA), where smooth muscle cells are replaced with amyloid deposits (Prior et al. 1996; Biffi and Greenberg 2011). These vessels are particularly susceptible to rupture when subjected to amyloid clearance stress (Agarwal et al. 2023; Chantran et al. 2019), allowing blood products to leak into the brain parenchyma via dose‐dependent BBB disruption (Pardridge 2020; Pedder et al. 2025), and a mechanism that parallels the linear relationship observed between plaque reduction and ARIA‐E in clinical trials (Pardridge 2020). Crucially, the high heterogeneity (*I* ^2^ = 92%) found in our pooled ARIA‐E safety data underscores the need for a highly cautious clinical interpretation. It indicates that the incidence of vasogenic edema fluctuates drastically between different study populations, heavily influenced by trial‐specific parameters such as explicit dosing protocols, titration schedules, or baseline patient vulnerabilities.

Beyond microvascular changes, treated patients experienced a significant, twofold increased risk of local injection site reactions. Conversely, broad systemic adverse events, including falls, nasopharyngitis, dizziness, back pain, diarrhea, and UTIs, occurred at statistically equivalent rates between the groups. While headache was reported frequently, its primary cause in these patients is localized neuro‐inflammation triggered by the antibody's mechanism of action (Bateman et al. 2023; Salloway et al. 2024). This chronic inflammatory state may affect overall immune function, potentially reducing resistance to opportunistic pathogens and causing upper respiratory or UTIs (Uettwiller et al. 2011). This confirms that gantenerumab's safety complications are predominantly “on‐target” phenomena tied directly to its intended perivascular amyloid‐clearing mechanisms rather than off‐target toxicities, highlighting the absolute necessity of rigorous patient screening and risk‐benefit monitoring in practice.

### 4.4 Clinical Implications

The findings of this updated meta‐analysis highlight critical considerations for the clinical use of gantenerumab in patients with AD. Rather than supporting routine or universal administration, these data dictate that any potential clinical application must rely on highly meticulous patient selection and specialized institutional infrastructure. Because the observed therapeutic benefits are modest and restricted to specific cognitive and functional endpoints, clinicians must carefully manage caregiver and patient expectations, emphasizing that the treatment may only subtly decelerate, rather than halt or reverse, symptom progression. More importantly, the high incidence of ARIA requires strict real‐world safety management protocols, including mandatory baseline genetic testing for ApoE ε4 status, careful exclusion of patients with advanced pre‐existing CAA, and structured serial MRI monitoring during the initial titration phases to identify and manage asymptomatic vasogenic edema or microhemorrhages before they progress to severe clinical events. This suggests that, based on current available clinical trial data, a clear therapeutic role for gantenerumab as a routine agent in early AD remains to be fully established. Clinicians should carefully weigh these limitations and potential risks when considering treatment options for patients with early AD.

### 4.5 Strengths and Limitations

This meta‐analysis is bounded by several limitations inherent to the original trial designs. Because data were analyzed at the trial level, individual balance among baseline characteristics could not be verified, and critical baseline parameters, specifically Expanded Disability Status Scale (EDSS) scores and total centiloid reductions, were completely absent. Furthermore, the included trials administered variable doses of gantenerumab without a paired subgroup analysis, presenting a potential confounding factor. This methodological divergence across the primary literature heavily drives the high statistical heterogeneity observed in both our amyloid‐PET (*I* ^2^ = 99%) and ARIA‐E (*I* ^2^ = 92%) datasets, which inherently limits our ability to draw a single, definitive clinical conclusion and reinforces the need for a more cautious interpretation of these specific pooled estimates. Missing or unpublished CDR‐SB data in the DIAN‐TU 2021 study restricted the pooled analysis.

Furthermore, while a publication bias assessment was successfully executed via funnel plot modeling for the CDR‐SB and ADAS‐Cog13 scores, these configurations are structurally constrained by the small total pool of eligible trials (*n* = 5 per outcome). Consequently, the low statistical power of asymmetry assessments means that localized selective reporting or unpublished small‐scale pilot registries cannot be entirely ruled out by visual diagnostics alone. Furthermore, while the inclusion of Google Scholar enhanced our search coverage for gray literature, its inherently fluid search algorithms may present minor challenges for long‐term replication, which we mitigated by enforcing strict screening thresholds. Additionally, the distinctive incidence of ARIA and injection site reactions introduces a high risk of functional unblinding, which could introduce bias into subjective cognitive and functional ratings by caregivers and clinicians. Finally, while the SCarlet RoAD study was terminated early, its primary endpoints failed to achieve statistical significance across all clinical tests after 2 years of evaluation, necessitating cautious interpretation. Future research must incorporate long‐term observational registries and subgroup analyses comparing explicit dosing regimens to clarify dose‐dependent safety boundaries and determine if prolonged amyloid clearance eventually yields meaningful cognitive benefits.

Balanced against these limitations, the study possesses clear methodological strengths that enhance the reliability of its findings. Restricting the inclusion criteria exclusively to RCTs ensures high‐quality evidence and minimizes confounding bias. The integration of advanced biomarkers, such as amyloid‐PET neuroimaging and cerebrospinal fluid assays (Aβ42, total tau, and phosphorylated tau), provides objective, verifiable evidence of biological target engagement. Finally, pooling data across multiple trials drastically enhance the statistical power required to detect subtle treatment effects and safety signals, while the high level of structural alignment and low heterogeneity across the included studies ensures a highly consistent and powerful synthesis of the existing global evidence base.

## 5 Conclusion

Although gantenerumab demonstrates biological target engagement by removing cerebral amyloid, the high statistical heterogeneity underlying both plaque clearance and ARIA‐E incidence mandates a highly cautious interpretation of its broader clinical profile. Its statistically significant benefits on individual functional (FAQ) and cognitive (ADAS‐Cog13) parameters remain consistently below accepted MCID thresholds. This clear gap confirms that minor statistical shifts do not scale up into widespread, clinically meaningful improvements for early‐stage Alzheimer's patients. Additionally, it significantly raises the risk of injection site reactions and amyloid‐related imaging abnormalities. Therefore, the current data does not fully justify its routine use in treating early‐stage AD. Furthermore, future research is warranted with a major focus on optimizing dose, patient selection, and combination strategies in order to improve safety and efficacy and provide significant clinical benefits.

## Author Contributions

M.N.J. conceptualized the study and formulated the research question. S.M.K. and J.M.H. conducted the literature search and screened studies. S.M. and A.M. performed the data extraction of the included studies. H.A. and A.M. performed the statistical analyses. M.K. assessed study quality and risk of bias. N.A., A.H., and A.Z. wrote the original manuscript. M.N.J. revised the manuscript for corrections.

## Funding

The authors have nothing to report.

## Ethics Statement

The authors have nothing to report.

## Consent

The authors have nothing to report.

## Conflicts of Interest

The authors declare no conflicts of interest.

## Supporting information




**Supplementary Figure**: brb371643‐sup‐0001‐FigureS1.docx

**Supplementary Table**: brb371643‐sup‐0002‐TableS1.docx

## Data Availability

All data referenced in this article are publicly available in the original publication and cited sources. No new data were generated or analyzed in the preparation of this manuscript.
