# Supplementary material for: Gantenerumab for Early Alzheimer's Disease: An Updated Systematic Review and Meta‐Analysis
Source: Brain Behav. 2026 Aug 16;16(8):e71643. doi: 10.1002/brb3.71643 (PMC13478553; doi:10.1002/brb3.71643)
Supplement: Supplementary file 1 [file BRB3-16-e71643-s001.docx]

| Study ID (Year) | Doi | Patient Population | Intervention | Reason for exclusion |
| --- | --- | --- | --- | --- |
| Boess et al. (2024) | 10.14283/jpad.2024.60 | Participants aged 50–90 with early symptomatic AD | Once-weekly subcutaneous gantenerumab up to 255 mg | Single-arm/ open-labelled trial with no randomized comparator or placebo |
| Bateman et al. (2025) | 10.1016/S1474-4422(25)00024-9 | Participants with dominantly inherited AD | Long-term high dose gantenerumab up to 1500 mg every 2 weeks | Open-label / single-arm (non-randomized) extension of DIAN-TU trial with a different population |
| Fillit et al. (2024) | 10.14283/jpad.2024.115 | Participants recruited from the GRADUATE Trial, age ≥66, with early symptomatic AD | None | Non-interventional linkage cohort of GRADUATE participants |
| Wagemann et al. (2024) | 10.1001/jamaneurol.2024.0991 | Dominantly Inherited AD gene-variant carriers | Gantenerumab 225 mg (subcutaneously every 4 weeks) and solanezumab 400 mg (intravenously every 4 weeks) | DIAN-TU biomarker trial with a different population and unmatched outcomes |
| Boada et al. (2025) | 10.1177/13872877241303644 | Participants from the Scarlet RoAD randomized trial (prodromal AD) | Subcutaneous gantenerumab, up titrated to up to 1200 mg Q4W | Uncontrolled open labelled extension (OLE) and re-uses participants from the Scarlet RoAD trial |
| Bittner et al. (2025) | 10.1002/alz.14414 | Participants with early AD | Gantenerumab (phase-3 dosing) | Reports biomarker analyses from the same GRADUATE I & II trials already included in previous meta-analysis |
| Salloway et al. (2025) | 10.1001/jamaneurol.2024.3937 | Early symptomatic AD patients with amyloid-beta pathology | Gantenerumab with 9-month uptitration | Post-hoc analyses and open-label extension data, which is non-randomized and not directly comparable with the previously included studies |
| Chen et al. (2025) | 10.1007/s00401-025-02890-7 | Participants with dominantly inherited AD | Gantenerumab and solanezumab | Histopathologic/autopsy analysis of 10 trial cases and therefore cannot be pooled with existing studies |

**Supplementary Table:** List of trials excluded from the meta-analysis.
