# Supplementary material for: Gantenerumab for Early Alzheimer's Disease: An Updated Systematic Review and Meta‐Analysis
Source: Brain Behav. 2026 Aug 16;16(8):e71643. doi: 10.1002/brb3.71643 (PMC13478553; doi:10.1002/brb3.71643)
Supplement: Supplementary file 2 [file BRB3-16-e71643-s002.docx]

**S1.** Risk of Bias (RoB) figure and summary
